# A New Invalid Dress

**Published:** 1890-10-04

**Authors:** 


### THERAPEUTICS.

### A NEW INVALID DRESS.

Most medical and surgical practitioners, nurses, and patients, have very frequently experienced much trouble and annoyance when dress has to be changed, and this in sickness is required very frequently. When changing the clothing, especially of helpless patients, this should be done without uncovering, and without raising the patient more than is necessary. Writers on the matter tell us that the dress should be taken from over each arm, and then drawn out from under the body. The arms should be placed in the sleeves of the clean garment, the body of which should be placed over the patient's head, and be drawn down without lifting the shoulders. All this, however, is not achieved without some difficulty. We have been shown a new dress for invalids, devised by Miss Shepherd, of 174, Brompton Road, which seems well calculated to reduce to a minimum the troublesomeness of the process of changing the clothes of a helpless patient. The upper part of the dress consists of a divided body; in fact, it consists of two parts. Each portion laps over the other back and front, where they may be secured if desired by either buttons or tapes. And each part buttons on the opposite side of the neck. The lower portion of the dress also consists of two parts, which encase the foot, leg, and thigh on each side. The advantages of such a dress are at once evident. Should a patient require massage only, that portion of the person to be manipulated may be exposed. Should part of the dress become soiled, only the soiled portion need be removed. Should any part of the person require surgical treatment, that part can be attended to without interference with the dress of other parts. Then as there is no slipping over the head necessary, there is no struggle to get the arms into the sleeves, neither need the patient even raise his arms to have the half coat put on. The garment may also be made so that the sleeves may be taken on and off, without removing the body part. Of course the garments as described above may be made of any material, and ornamented in any manner which may be desired. We think Miss Shepherd's divided dress for invalids will be extensively adopted.

